# Metabolomic Profile, Plasmatic Levels of Losartan and EXP3174, Blood Pressure Control in Hypertensive Patients and Their Correlation with COVID-19

**DOI:** 10.3390/ph16091290

**Published:** 2023-09-13

**Authors:** Kamila A. Queiroz, Everton P. Vale, Manuel Martín-Pastor, Lílian G. S. Sólon, Francisco F. O. Sousa

**Affiliations:** 1 Graduate Program on Pharmaceutical Sciences, Department of Biological & Health Sciences, Federal University of Amapa, Macapa 68903-419, Brazil; kamila_queiroz2008@hotmail.com (K.A.Q.); liliansolon@yahoo.com.br (L.G.S.S.); 2 Laboratory of Quality Control, Bromatology and Microbiology, Department of Biological & Health Sciences, Federal University of Amapa, Macapa 68903-419, Brazil; everton.pantoja.vale@gmail.com; 3 Graduate Program on Pharmaceutical Innovation, Department of Biological & Health Sciences, Federal University of Amapa, Macapa 68903-419, Brazil; 4 Unidade de Resonancia Magnetica, Área de Infraestruturas de Investigación, Campus Vida, Universidad de Santiago de Compostela, 15782 Santiago de Compostela, Spain; manuel.martin@usc.es

**Keywords:** hypertension, COVID-19, metabolomics, losartan, EXP3174, plasmatic level

## Abstract

Systemic arterial hypertension (SAH) is one of the most prevalent chronic diseases worldwide and is related to serious health complications. It has been pointed out as a major risk factor for COVID-19. This study aimed to determine the impact of COVID-19 on the metabolomic profile, the correlation with the plasmatic levels of losartan and its active metabolite (EXP3174), biochemical markers, and blood pressure (BP) control in hypertensive patients. ^1^H NMR metabolomic profiles of hypertensive and normotensive patients with and without previous COVID-19 diagnosis were identified. Plasmatic levels of LOS and EXP3174 were correlated with BP, biochemical markers, and the metabolomic fingerprint of the groups. Biomarkers linked to important aspects of SAH and COVID-19 were identified, such as glucose, glutamine, arginine, creatinine, alanine, choline, erythritol, homogentisate, 0-tyrosine, and 2-hydroxybutyrate. Those metabolites are indicative of metabolic alterations, kidney damage, pulmonary dysfunction, and persistent inflammation, which can be found in both diseases. Some hypertensive patients did not reach the therapeutic levels of LOS and EXP3174, while the BP control was also limited among the normotensive patients with previous COVID-19 diagnoses. Metabolomics proved to be an important tool for assessing the effectiveness of losartan pharmacotherapy and the damage caused by SAH and COVID-19 in hypertensive patients.

## 1. Introduction

The new coronavirus 2019 (COVID-19), caused by SARS-CoV-2 (severe acute respiratory syndrome Coronavirus 2), emerged in Wuhan in December 2019 and spread globally, causing global public health and economic concerns [1,2].

It is an enveloped virus formed by single-stranded, positive-sense RNA, which recognizes angiotensin-converting enzyme 2 (ACE2) as the functional cell entry receptor. ACE2 belongs to the angiotensin-converting enzyme (ACE) family and plays an important role in human physiological functions, especially in BP control [2,3,4].

SAH is a common clinical condition characterized by a sustained increase in BP and is considered an important risk factor for cardiovascular diseases, including coronary heart disease, stroke, and heart failure [5]. COVID-19 triggered great global concern due to its significant impact on public health and its serious complications. Recent studies suggest a possible association between hypertension and the severity of COVID-19 infection [2], where hypertension may predispose individuals to greater susceptibility to SARS-CoV-2 infection and serious complications. Therefore, understanding the relationship between the metabolomic profile, plasma levels of losartan and its active metabolite (EXP3174), blood BP and COVID-19 may provide important insights for the clinical management of these patients [6].

In recent years, there has been a growing interest in understanding the metabolomic profile of hypertensive patients as an important tool for diagnosis, risk stratification, and development of personalized therapies. Metabolomics is a scientific approach that seeks to identify and quantify the metabolites present in a biological sample, providing a comprehensive view of the individual metabolic state [7].

Recent data describe the general clinical aspects and epidemiological characteristics of patients with COVID-19, and many reports point out that cardiac injury is related to a higher risk of mortality in those patients [1,8].

The pathophysiological mechanisms of acute COVID-19 are direct viral infection, endothelial and microvascular injury, compromise of the immune system and stimulation of a high inflammatory state, and maladaptation of the angiotensin II-converting enzyme pathway [9,10].

Moreover, the main hypothesis for the different levels of manifestations is related to the expression of ACE2 in alveolar epithelial cells. As the lungs are still developing in childhood, the expression of this enzyme is reduced and may correspond to a protective factor for severe disease. In addition, the maturity and functionality of this extracellular component are reduced in children [11].

The difference in the projection of these receptors is also observed between genders and ethnicities, with men and Asians having more ACE2 in their alveolar cells compared to women and other ethnicities, respectively, which could explain worse outcomes in these groups [12].

Individual differences may arise from pathophysiological conditions, diet, environment, and genes, all of which affect drug metabolism and disposition. Pharmacotherapeutic treatments are challenging due to the variability of the pharmacological response between individuals [13].

Among the therapeutic options, angiotensin-converting enzyme inhibitors (ACEIs) and angiotensin II receptor blockers (ARBs) are suppressive agents of the renin–angiotensin–aldosterone system, considered first-line drugs in the management of hypertensive patients [6,14].

These drugs work in a contradictory way, as they decrease the levels of angiotensin II and, consequently, the cytokines induced by it, at the same time promoting hyperexpression of ACE2 receptors [14,15].

Losartan (2-butyl-4-chloro-1-{[2′-(1H-tetrazol-5-yl) biphenyl-4-yl]methyl}-1H-imidazol-5-yl)methanol) is a functionalized imidazole molecule with a potent antagonism of angiotensin II receptor subtype 1. It is a total, competitive, and specific antagonist of ACE2 receptors, widely used in the treatment of hypertension and congestive heart failure [16,17]. It was the first orally available angiotensin receptor antagonist without agonist properties. This category has the ability to block the angiotensin II receptor, resulting in vasodilation and a reduction in blood pressure. These drugs are effective in controlling high blood pressure in hypertensive patients [18].

After oral administration, losartan is rapidly absorbed, reaching the peak concentration 1 to 2 h after administration. Approximately 14% of the dose is converted to the pharmacologically active metabolite EXP3174, which is 10 to 40 times more potent, with an estimated half-life ranging from 6 to 9 h. The pharmacokinetics of losartan and EXP3174 are linear and dose-dependent and do not change significantly with repeated administration [19].

Recently, some pharmacokinetic parameters (AUC, C_max_, and T_max_) of losartan have been associated with the plasma pharmacometabolomic profile [20], which can be used to support clinical decisions [21].

Accordingly, this study aimed to determine the impact of COVID-19 on the plasma metabolomic profile of hypertensive patients, the effects of plasma levels of losartan and its active metabolite (EXP3174) on BP control, and the possible association between hypertension and the severity of COVID-19 infection. Understanding these relationships may pave the way for more personalized therapeutic strategies and preventive interventions to minimize the risks associated with SAH and COVID-19. In addition, personalized medicine has become increasingly important in clinical care to improve efficacy and reduce drug-related problems.

## 2. Results

### 2.1. Patients Characteristics

Based on the criteria defined, patients were divided into four groups: hypertensive COVID-19 positive (HCP), normotensive COVID-19 positive (NCP), hypertensive COVID-19 negative (HCN), and normotensive COVID-19 negative (NCN).

For the groups of hypertensive patients, subdivisions were made based on the different periods from the COVID-19 diagnosis, according to the following criterion: diagnosis below 3 months: HCP 3 months; 3-to-6-months diagnosis: HCP 6 months; and diagnosis over 6 months: HCP 1 year. Normotensive patients with a positive diagnosis of COVID-19 were subdivided in the same way, forming the subgroups NCP 3 months, NCP 6 months, and NCP 1 year, respectively (Table 1).

The clinical variables obtained from the patients were analyzed, and the most relevant data found are listed in Table 2.

The BP of hypertensive patients was measured at three different times (before, 1.5 h, and 3 h after losartan administration) in order to assess the response to the administration of losartan, while for normotensive patients, it was measured only at the first moment. Most hypertensive patients initially presented elevated BP values (55 and 58% for HCP and HCN, respectively), which gradually decreased after losartan administration (down to 20 and 41% for HCP and HCN, respectively). Moreover, BP was also found to be above normal clinical levels in 28% of the normotensive patients with a previous diagnosis of COVID-19 (Table 2).

Most patients were vaccinated with at least the first dose of one of the approved COVID-19 vaccines, as during the collection period, only the first dose was available. Relevant biochemical alterations were noticed, especially in the hypertensive patients with a previous diagnosis of COVID-19 (HCP group): LDH (lactate dehydrogenase) (25%), glucose (75%), and CRP (C-reactive protein) (55%), while the HCN group presented a limited number of patients with those alterations: 5.8, 35 and 23.5%, respectively. It is also important to observe the high glucose levels found in the group of normotensive patients with a previous diagnosis of COVID-19 (NCP) (61.1%).

### 2.2. Plasmatic Quantification of Losartan and EXP3174

The plasmatic quantification of losartan and its metabolite was performed according to the method described by Santos et al., 2023 [22]. The calibration curves obtained from the bioanalytical phase showed a strong linear relationship, with R² values greater than 0.99 for both analytes, with very low limits of detection and quantification of losartan (LoD 0.5 ng/mL, LoQ 5 ng /mL) and EXP3174 (LoD 2 ng/mL, LoQ 5 ng/mL), confirming the high sensitivity of the method for quantification of losartan in human plasma. The method presented satisfactory precision, with the coefficient of variation (CV) values between 1.60 and 19.02% and accuracy with values ranging from 43.80 to 579.07%.

**Figure 1:**
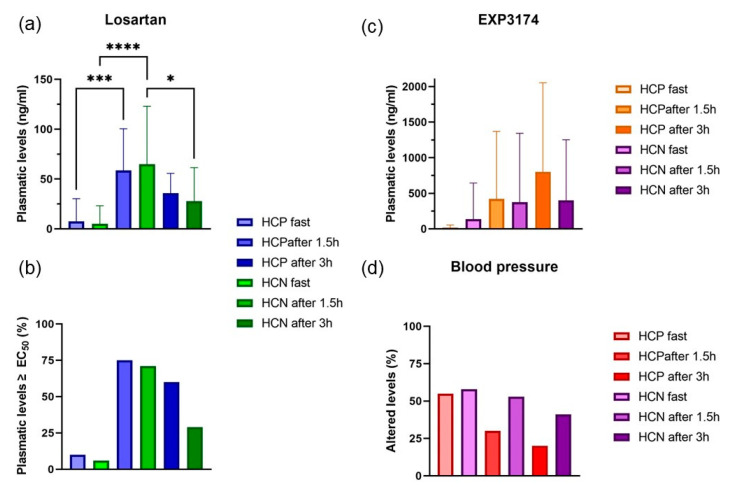
Plasmatic levels of (**a**) losartan, (**b**) percentage of patients who reached the EC_50_ (32 ng/mL), (**c**) plasmatic levels of EXP3174, and (**d**) percentage of patients with altered blood pressure levels at fasting after 1.5 h and 3 h after the oral administration of losartan tablets (detailed information can be found in Table S1). *, *** or **** mean *p* < 0.05, *p* < 0.001, and *p* < 0.0001 statistical significance, respectively, after ANOVA followed by Tukey’s post-test.

According to the results, a limited number of hypertensive patients, both with (HCP) and without (HCN) previous COVID-19 diagnosis, were found to have their BP levels controlled (Figure 1d). At first, the percentage of hypertensive patients with BP under control between the HCP and HCN groups was very similar (Figure 1d), while the mean plasmatic levels of losartan (Figure 1a,b) and EXP3174 (Figure 1c) were found below the effective levels (Table S1). Nonetheless, after 1.5 and 3 h, the patients of the HCN group were found to be more responsive to the anti-hypertensive effects of losartan and EXP3174 (Figure 1). Even if the number of patients in the HCN group who reached the plasmatic levels of losartan (Figure 1a) and EXP3174 (Figure 1c) above the EC_50_ was smaller (Table S1), the percentage of patients in this group who reached BP control was far higher than those in the HCP group: fast (55 vs. 58%), after 1.5 h (53 vs. 30%), and after 3 h (41 vs. 20%), respectively (Figure 1d). Therefore, the previous COVID-19 infection may have affected the metabolism of losartan. Bearing in mind that the valley levels were reduced in both groups and after the supervised administration of losartan, a limited number of patients reached adequate BP control, even if the plasmatic levels overcame the minimum reported concentration. This aspect is even more relevant for EXP3174, as its C_max_ is expected to occur at least 3 h after administration [22], and in both groups (Figure 1b), it was mostly reached. Nonetheless, the number of patients with BP levels within the normal range did not follow the same pattern. This finding reinforces the fact that a considerable number of normotensive patients with previous COVID-19 diagnosis, NCP (28%), presented BP values above the normal range (Table 2).

#### NMR Metabolomics

Nuclear magnetic resonance (NMR) metabolomics based on fingerprint analysis and segmentation [21,23] or ¹H_T2 was used. Potential biomarkers related to SAH and COVID-19 were identified.

The data that demonstrated significance after satisfying the PCA-based spectral quality criterion and leading to favorable separation of groups by multivariate and univariate analysis were selected to assess, first, differences between groups and also in pairs, as follows.

### 2.3. HCP Groups

#### 2.3.1. HCP vs. NCP Groups

**Figure 2:**
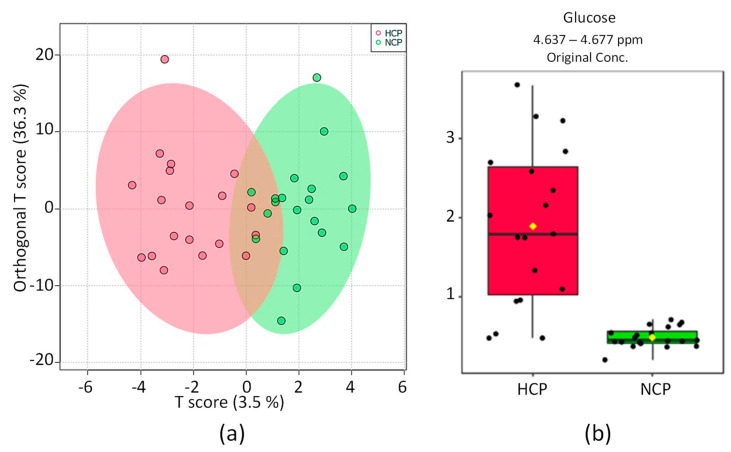
Directed analysis statistics of ¹H_T₂ spectra of plasma samples from HCP group (in red) vs. NCP group (in green). (**a**) OPLS-DA score chart (*n* = 39, R^2^ = 0.744, and Q^2^ = 0.0906, *p* = 0.03). (**b**) Bar graphs of the distribution of NMR intensities of glucose (*n* = 39, *t*-test *p* < 3 × 10^−7^).

The distribution of the OPLS-DA score graph for the HCP and NCP groups (Figure 2a) shows a small overlap between the groups. Nonetheless, a significant difference in their metabolomic profiles was found. The discriminative metabolite was glucose (Figure 2b). The 2D scoring axes cover 3.5% and 33.6% of the total variability.

The univariate analysis of the NMR integral in the buckets corresponds to the elevated glucose metabolite for hypertensive patients who had a previous diagnosis of COVID-19.

#### 2.3.2. HCP 3 Months vs. 6 Months vs. 1 Year

**Figure 3:**
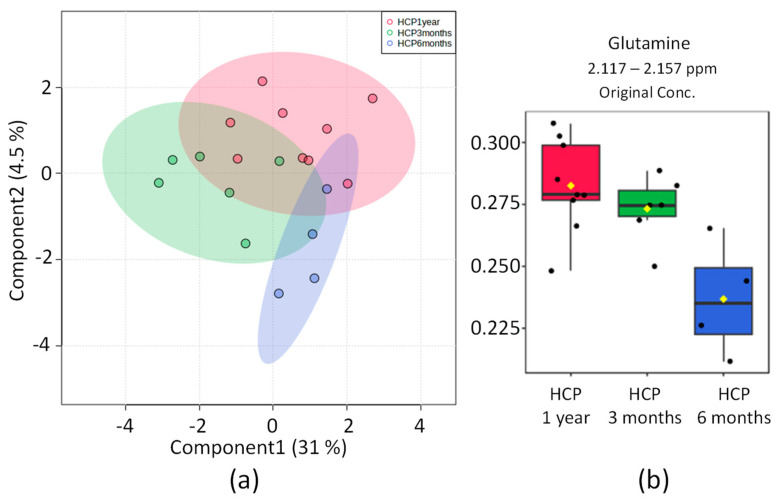
Statistics of the directed analysis of ¹H_T₂ spectra of plasma samples from the HCP 1-year (in red), HCP 6 months (in blue), and HCP 3 months (in green) groups. (**a**) Sparse PLS-DA score chart (*n* = 19, separation distance *p* = 0.796). (**b**) Bar graphs of the distribution of NMR intensities of identified metabolites with differences between the three groups: glutamine (*n* = 19, ANOVA *p* = 0.013).

The hypertensive patients were subdivided according to the time lapse after COVID-19 diagnosis. Upon OPLS-DA analysis, one region in the spectrum with maximum discrimination power corresponding to glutamine metabolites was found. Nonetheless, the score graph in Figure 3b shows a partial overlap between groups, and the 2D scoring axes cover 31% and 4.5% of the total variability.

The most significant metabolomic difference found between the three diagnostic periods for COVID-19 in hypertensive patients was glutamine, which showed a tendency to be elevated in the subgroup HCP 3 months.

#### 2.3.3. HCP 3 Months vs. HCP 1 Year

**Figure 4:**
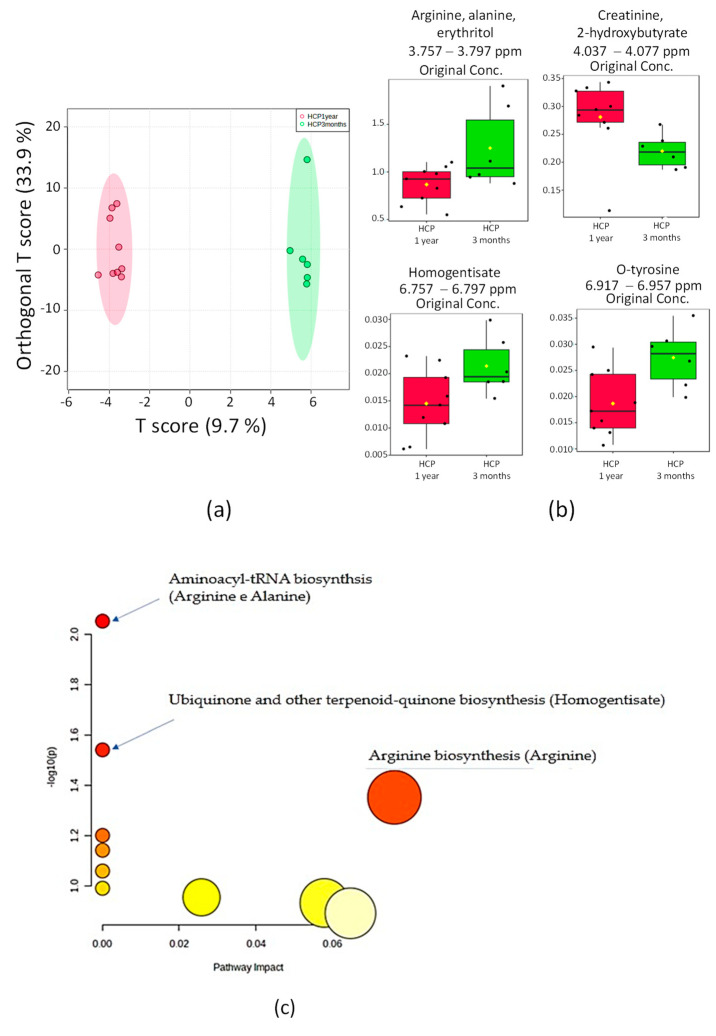
Directed analysis statistics of ¹H_T₂ spectra of plasma samples from HCP 3 months vs. HCP 1 year. (**a**) OPLS-DA score chart (*n* = 16, R^2^ = 0.888 and Q^2^ = −0.206, *p* = 0.2). (**b**) Bar graphs of normalized peak intensities of the identified metabolite with differences between the two groups. From left to right, the respective value of *p* of the *t*-test is 0.04, 0.02, 0.09, and 0.02. (**c**) Metabolic topology pathway results based on potential metabolites in OPLS-DA models.

A remarkable difference was found between the groups with recent COVID-19 (3 months) and long COVID-19 (1 year) (Figure 4a). OPLS-DA found a variation in relevant buckets corresponding to arginine, alanine, erythritol, creatine, 2-hydroxybutyrate, homogentisate, and o-tyrosine (Figure 4b).

A significant difference was observed between the two subgroups of hypertensive patients according to the time lapse after COVID-19. The metabolites arginine, alanine, erythritol, homogentisate, and o-tyrosine were found elevated in the HCP 3 months group, while creatinine and 2-hydroxybutyrate were found more elevated in the HCP 1 year group.

A total of seven metabolites selected as most relevant were loaded for pathway analysis and hypergeometric testing. According to the analysis, the metabolism of alanine + arginine (*p* = 0.0088499; FDR = 0.74339), the homogentisate biosynthesis (*p* = 0.028734; FDR = 1.0), and the arginine metabolism (*p* = 0.044409; FDR = 1.0) were the most significant pathways correlated between the groups HCP 3 months and HCP 1 year (Figure 4c).

#### 2.3.4. HCP 1 Year vs. NCP 1 Year

**Figure 5:**
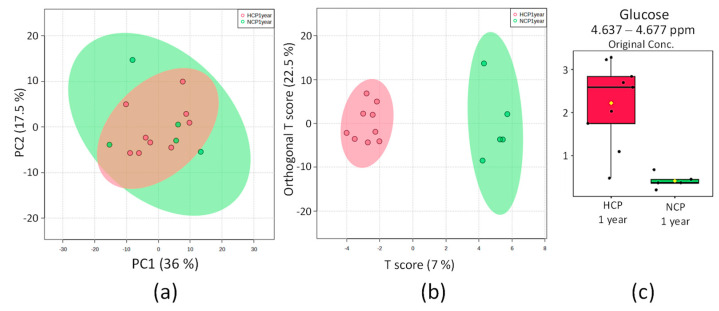
Statistics of the directed analysis of ¹H_T₂ spectra of plasma samples from the HCP 1 year (in red) and NCP 1 year group (in green). (**a**) PCA score chart. (**b**) OPLS-DA score graph (*n* = 14, R^2^ = 0.982, and Q^2^ = 0.127, *p* = 0.07). (**c**) Bar graphs of the distribution of NMR intensities of glucose, the only metabolite with differences between the two groups (*n* = 14, *t*-test *p* = 4 × 10^−4^).

By comparing the COVID-19 longa hypertensive and normotensive patients, the distribution of the PCA score for the two groups (Figure 5a) is highly compact and relatively random, with no obvious outliers to be considered. OPLS-DA found regions in the spectrum (buckets) with discriminating power that could correspond to glucose. The scoring chart in Figure 5b shows full separation between the groups, and the 2D scoring axes cover 7% and 22.5% of the total variability.

#### 2.3.5. HCP 6 Months vs. NCP 6 Months

**Figure 6:**
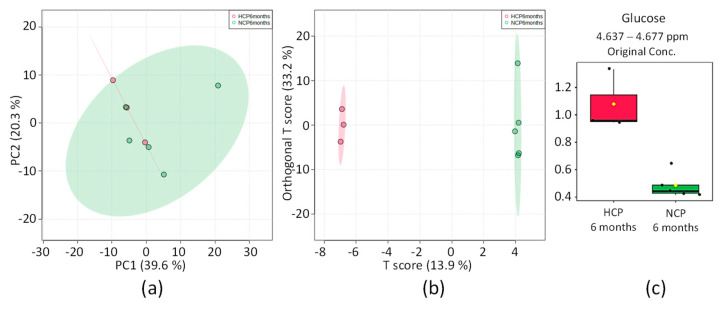
Statistics from the directed analysis of ¹H_T₂ spectra of plasma samples from the HCP 6 months (in red) vs. NCP 6 months (in green). (**a**) PCA score chart, (**b**) OPLS-DA score chart (*n* = 8, R^2^ = 0.1425, and Q^2^ = 0.33, *p* = 0.14). (**c**) Bar graph of the distribution of NMR intensities of glucose, metabolite identified with difference between the two groups (*n* = 8, *t*-test *p* = 0.002). Its bucket integrals in the spectra were considered relevant for the OPLS-DA classification obtained in (**b**).

The distribution of the PCA score graph for the two groups (see Figure 6a) is also highly compact and relatively random, with no obvious outliers to consider. OPLS-DA found five regions in the spectrum (buckets) with maximum discrimination power corresponding to the metabolite glucose. The scoring chart in Figure 6b shows excellent group separation with no overlap, and the 2D score axes cover 13.9 and 33.2% of the total variability.

#### 2.3.6. HCP 3 Months vs. NCP 3 Months

**Figure 7:**
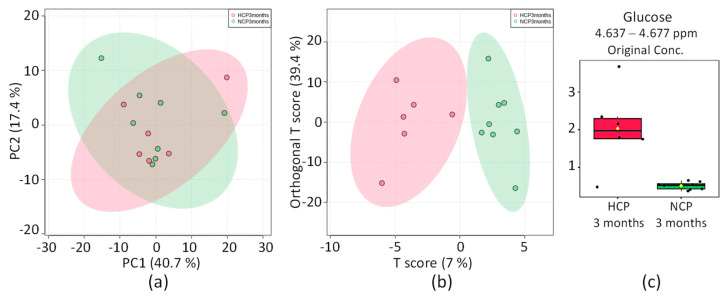
Statistics of directed analysis of ¹H_T₂ spectra of plasma samples from the HCP 3 months (in red) vs. NCP 3 months (in green) groups. (**a**) PCA score chart. (**b**) OPLS-DA score graph (*n* = 14, R^2^ = 0.88, and Q^2^ = 0.09, *p* = 0.33). (**c**) Bar graphs of normalized peak intensities of glucose, identified as the only metabolite with difference between the two groups (*n* = 14, *t*-test *p* = 0.015). Its bucket integrals in the spectra were considered relevant for the OPLS-DA classification obtained in (**b**).

The distribution of the PCA score graph among the groups of hypertensive and normotensive patients with a more recent COVID-19 diagnosis (<3 months) (Figure 7a) is also highly compact and relatively random, with no obvious outliers to consider. OPLS-DA found a region in the spectrum (buckets) with the maximum discriminating power corresponding to b-Glc metabolites (Figure 7c). The score plot in Figure 7b shows the split between the HCP 3 months and NCP 3 months groups without overlapping, while the 2D score axes cover 7% and 39.4% of the total variability.

The univariate analysis of the NMR integral in the buckets corresponds to glucose, which was also found elevated for hypertensive patients who contracted COVID-19 more recently. Glucose is part of the metabolic pathway that triggers inflammatory immune responses in the infection caused by COVID-19, and according to the HCP vs. NCP analysis, it was found recurrent for the hypertensive patients who contracted COVID-19, despite the time lapse undergone.

### 2.4. RMN of NCP Groups

In order to check the role of COVID-19 in the normotensive patients and contrast to the data obtained from the hypertensive patients, those patients were also checked according to the time lapse after COVID-19, including one analysis between the 3 subgroups (NCP 3 months, 6 months, and 1 year) and also an analysis between the two extremes (NCP 3 months and 1 year).

#### 2.4.1. NCP 3 Months vs. NCP 6 Months vs. NCP 1 Year

**Figure 8:**
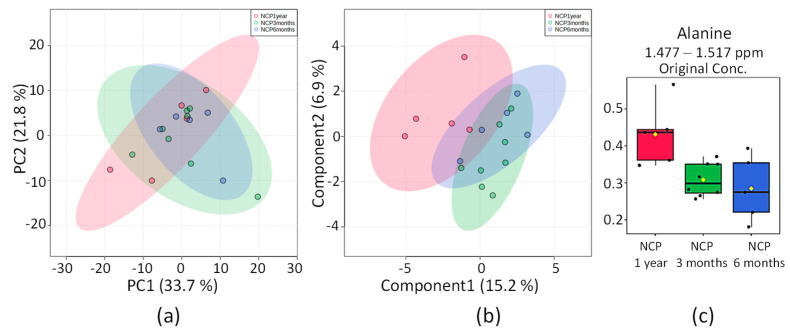
Statistics of the directed analysis of ^1^H_T_2_ spectra of plasma samples from the NCP 3 months (in green), NCP 6 months (in blue), and NCP 1-year (red) groups. (**a**) PCA score chart. (**b**) Sparse PLS-DA score graph (separation distance *p* = 0.575). (**c**) Bar graphs of the distribution of NMR intensities of alanine, found significantly different within the 3 groups (*n* = 18, ANOVA *p* = 0.04).

The distribution of the PCA score graph for the three groups (Figure 8a) is highly compact and relatively random, with no obvious outliers to consider. OPLS-DA found a region in the spectrum (buckets) with maximum discrimination power corresponding to alanine. The score graph in Figure 8b shows partial overlap among the three groups, with the NCP 6 months group coherently between the NCP 3 months and NCP 1-year groups, while the 2D scoring axes cover 15.2% and 6.9% of the total variability.

Alanine was found significatively different among the normotensive patients divided into 3 periods after COVID-19 diagnosis. It was found remarkably more elevated in the subgroup NCP 1 year.

#### 2.4.2. NCP 3 Months vs. NCP 1 Year

**Figure 9:**
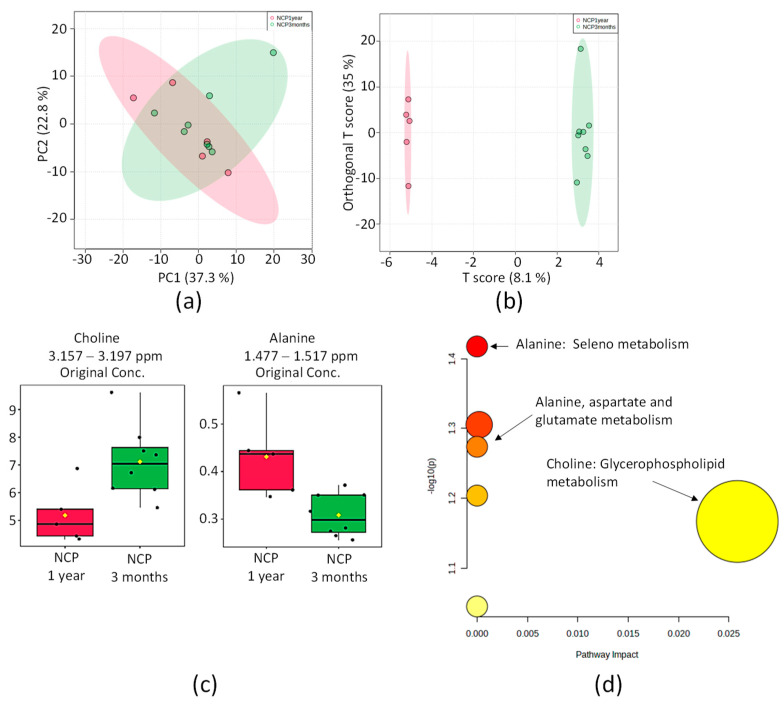
Statistics of the directed analysis of ¹H_T₂ spectra of plasma samples from the NCP 3 months (in green) vs. NCP 1-year (in red) groups. (**a**) PCA score chart. (**b**) OPLS-DA score graph (*n* = 13, R^2^ = 0.999, and Q^2^ = 0.448, *p* = 0.054). (**c**) Bar graphs of normalized peak intensities of identified metabolites with differences between the two groups. Its bucket integrals in the spectra were considered relevant for the OPLS-DA classification obtained in (**b**). From left to right, the respective *t*-test value of *p* is 0.018 and 0.018. (**d**) Metabolic topology pathway results based on potential metabolites in OPLS-DA models.

By comparing solely, the two extremes, the distribution of the PCA score graph for the NCP 3 months and NCP 1-year groups (Figure 9a) does not show overlap (Figure 9b), and a significant difference in the metabolomic profile between these groups was found, based on the discriminative metabolites choline and alanine. The 2D score axes cover 7.9% and 35% of the total variability.

From the OPLS-DA analysis of the buckets, two metabolites were identified as the most relevant: choline and alanine. Univariate analysis of the NMR integral on the buckets corresponding to these metabolites is displayed in Figure 9c.

According to the analysis, the metabolism of seleno (*p* = 0.038237; FDR = 1.4175) and the alanine, aspartate, and glutamate metabolism (*p* = 0.053254; FDR = 1.2736) were the most significant pathways correlated between the NCP 3 months and NCP 1 year (Figure 9d).

#### 2.4.3. HCN vs. NCN Groups

**Figure 10:**
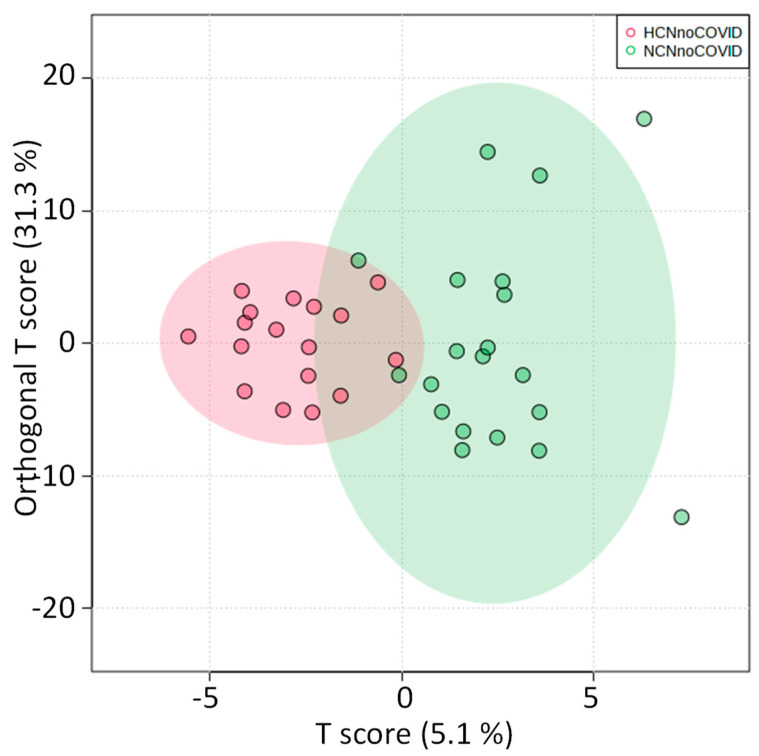
Statistics of the directed analysis of ¹H_T₂ spectra of plasma samples from HCN (in red) vs. NCN (in green) groups. OPLS-DA score chart (*n* = 37, R^2^ = 0.716, and Q^2^ = 0.286, *p* = 0.00115).

To check the role of hypertension in the metabolomic analysis above, we compared the two groups of patients without previous COVID-19 diagnosis (HCN and NCN, hypertensive and normotensive, respectively). The distribution of the OPLS-DA score graph did not show any region in the spectrum (buckets) with discriminating power that could correspond to metabolomic findings. The scoring chart in Figure 10 shows a partial overlap between groups, and the 2D scoring axes cover 5.1% and 31.3% of the total variability.

In the univariate analysis of the NMR integral in the buckets, no considerable metabolomic differences were observed between the groups, which may indicate that these metabolomic disorders found among the other pairs are not related to hypertension but mainly to COVID-19.

### 2.5. Correlation Maps

**Figure 11:**
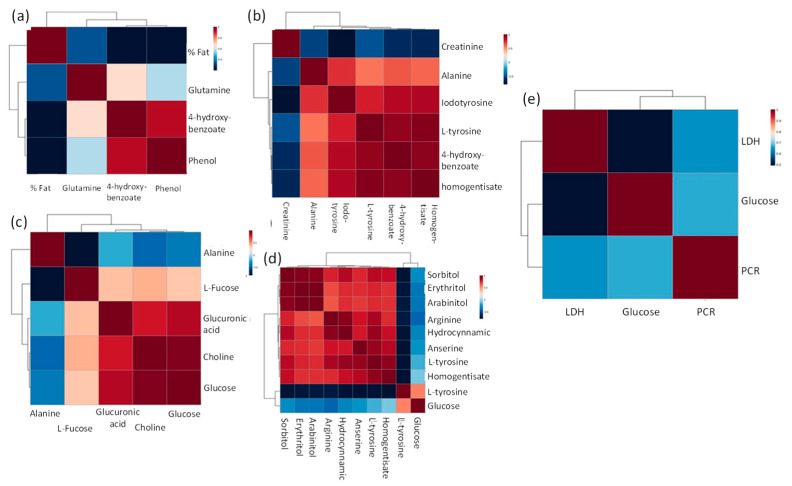
Comprehensive profile of metabolites identified by CE-TOFMS analysis in plasma was analyzed by hierarchical clustering. Grouping method: UPGMA (unweighted average); similarity measure: Euclidean distance; ranking function: mean value. The heat map shows the base 2 logarithmic ratio to the means of the corresponding compared groups. (**a**) Correlation map between HCP 3 months and HCP 6 months and HCP 1 year. (**b**) HCP correlation 3 months vs. HCP 1 year. (**c**) NCP 3 months vs. NCP 1 year. (**d**) correlation of HCP 6 months vs. NCP 6 months, and (**e**) correlation between HCP 3 months and NCP 3 months.

Figure 11 shows a representation of the heat map of the metabolomic profiles, with a hierarchical grouping of the quantified metabolites in the samples for groups in pairs and trios correlated with clinical variables, such as BP, plasma levels of losartan, and biochemical markers. Glucose showed good correspondence in both biochemical and metabolomics analyses, validating the results obtained.

## 3. Discussion

^1^H NMR plasma metabolomics were correlated with the biochemical parameters, BP, and plasmatic levels of losartan and EXP3174. According to the patients’ plasma analysis, 40.5% reached the minimum therapeutic concentration of EXP3174, whose 40% were of the HCP and 35.3% of the HCN groups. The remaining patients did not reach therapeutic levels of losartan or EXP3174 before, 1.5 h, and 3 h after oral administration. For those patients, the dose of losartan should be adjusted so that the minimum therapeutic dose is reached, and as a result, the therapeutic effect can be achieved.

In both groups, HCP and HCN, some patients who reached the therapeutic concentration of EXP3174 still had high BP levels, 10% from the HCP and 17.6% from the HCN. This is an important finding, considering that uncontrolled SAH is a predisposing factor for several health problems, such as renal injury and subsequent chronic kidney disease. Therefore, it is prudent for these patients to have their therapeutic regimen revised so that the clinical effect of the anti-hypertensive treatment is achieved. No significant differences regarding the pharmacokinetics and pharmacodynamics of losartan and EXP3174 were attributed to COVID-19; even so, BP control was affected.

In addition, it was observed that 27% of the NCP group had high BP values, indicative of SAH, a fact that may be related to the metabolic changes caused by COVID-19 or hypothetically non-diagnosed patients, the last being less reasonable as no altered levels of BP between the patients in the group NCN, i.e., normotensive without COVID-19 previous diagnosis, was found (Table 1). This is an alarming factor, as this group was expected to have normal BP values.

When analyzing the biochemical markers, high levels of CRP were found in 55% of patients in the HCP group, 23.5% of patients in the HCN group, 5.6% of patients in the NCP group, and 20% of patients in the NCN group. This may be related to the fact that CRP is an indicator of cardiovascular risk, inflammation and endothelial dysfunction, and vascular and renal injury [24], in addition to the fact that it is found to be rapidly increased after a positive diagnosis of COVID-19 [25]. Nonetheless, considering the COVID-19 longa found among the volunteers, it is still a very remarkable aspect.

One of the most prevalent factors related to COVID-19 progression is hyperglycemia [26]. Elevated and uncontrolled blood glucose levels for a prolonged period [27] due to insulin resistance [28] and impaired insulin production caused by COVID-19 are commonly found [29]. According to the results of metabolomics and biochemical tests, a tendency towards increased blood glucose in patients of the HCP group, when compared to the groups of NCP patients, was found, which may indicate that both hypertension and COVID-19 are factors that contribute to the dysregulation of glucose levels.

L-Glutamine is the most abundant amino acid in the blood, released mainly from the skeletal muscles and transported to a variety of tissues [30]. Although most tissues can synthesize, during periods of stress, demand outstrips supply, and expression levels of glutamine transporters in plasma membranes become critical [30]. Glutamine is essential for T cell differentiation and macrophage polarization, and high levels can increase oxygen consumption in effector T cells and promote T cell activation [31]. Glutamine was found to be elevated and positively correlated with C-reactive protein (CRP) levels in patients after COVID-19, suggesting that inflammation persists even in recovered patients [32]. For instance, it was found to be comparatively elevated in the HCP 3 months group, followed by HCP 6 months and HCP 1 year, which may be related to the persistence of inflammation and dysregulation of post-COVID protein metabolism.

Elevated arginine levels to cellular immune status or airway remodeling in patients recovered from COVID-19 with abnormal lung carbon monoxide (CO) diffusion capacity has been reported [33]. The data showed that the HCP 3 months group also presented higher levels of arginine when compared to the HCP 1 year group, which may indicate the recovery of post-COVID-19 lung functions.

Alanine was found lower in the NCP 3 months group when compared to the NCP 6 months and NCP 1-year groups. In this context, alanine becomes important as a glycogen component of the Cahill cycle (also known as the glucose–alanine cycle) to meet energy requirements [34]. Changes in alanine levels have important effects on muscle integrity; for example, reduced levels of alanine reduce the biosynthesis of carnosine, an important dipeptide that protects against muscle wasting associated with sarcopenia [35], which can be one of the aspects found in COVID-19 longa [36].

Furthermore, patients with COVID-19 often have higher energy requirements, skeletal muscle catabolism, and sarcopenia, which may increase the risk of death [36]. Therefore, maintaining alanine homeostasis is critical in order to reduce the risk of severe disease [36,37]. Interventions such as dietary alanine supplementation can reverse these deleterious effects of SARS-CoV-2 [38]. However, when we compare the HCP groups, this tendency is reversed, being higher in the HCP 1 year group than in HCP 3 months. It may be possible that SAH may have a certain influence on the alanine levels during COVID-19 recovery.

Erythritol is a sugar alcohol (or polyol) occurring widely in nature and is used as a food additive and sugar substitute in several foods, including wine, sake, beer, watermelon, pear, grape, and soy sauce [39]. Its accumulation may be related to uremia, where uremic toxins accumulate due to the inability or reduced capacity for renal clearance, a very common condition in hypertensive and diabetic patients [40], which may be an early indication of kidney dysfunction.

Kidney injury is a common complication of COVID-19 and leads to the accumulation of toxic metabolites for other systems [41]. Some authors reported in their study that creatinine levels, an indicator of kidney function, were higher in recovered and moderate and critical patients with COVID-19 than in healthy patients, indicating that post-COVID kidney damage cannot be fully reversed [42]. These values remained high in COVID-19 patients at all sampling times, which may be related to dehydration due to strong inflammation; however, renal failure cannot be excluded. Interestingly, this tendency did not change over time, and patients in the post-acute phase showed a remaining increased plasmatic level of creatinine.

The same tendency was observed in the metabolomics results when comparing the HCP 1 year and HCP 3 months groups; in the first group, creatinine values remained high, indicating persistent kidney damage, which may also have a predisposition combined with the picture of SAH presented by those patients.

2-Hydroxybutirate, also known as 2-Hydroxybutyric acid and alpha-hydroxybutyrate, is an organic acid derived from alpha-ketobutyrate. Alpha-ketobutyrate is produced by amino acid catabolism (threonine and methionine) and glutathione anabolism (cysteine formation pathway) and is metabolized into propionyl-CoA and carbon dioxide [43]. Recently it has been noted that elevated levels of 2-hydroxybutyrate in the plasma is a good marker for early-stage type II diabetes [44]. Its elevation in HCP 1-year patients may be related to the increased levels of blood glucose observed in groups with a positive diagnosis for COVID-19, as the glucose metabolism is consistently affected [45], despite the time lapse considered.

Homogentisate is one of the six enzymes that participate in the degradation of phenylalanine and tyrosine. Its accumulation causes insoluble ochronotic pigments to be deposited in the connective tissues, resulting in degenerative arthritis [46]. Its increase in patients in the HCP 3 months group, when compared to the HCP 1 year group, may be related to the metabolic changes that occur during COVID-19.

o-Tyrosine is a normal human metabolite. Its presence is possible due to the hydroxylation of l-phenylalanine by the hydroxyl radical (*OH), and it is proposed as a hydroxy radical biomarker of oxidative damage to proteins [47]. Its elevation in the HCP 3 months group compared to the HCP 1 year may also be associated with the metabolic dysregulation that occurs during COVID-19.

Choline is an essential nutrient required for many important physiological functions, including methyl group metabolism, structural integrity, and cell signaling [48,49]. It was detected that the NCP 1 year group presented lower levels of choline when compared to NCP 3 months, which may be related to the recovery from COVID-19.

High plasmatic levels of choline are a cardiometabolic risk factor and are associated with a history of cardiovascular disease in elderly individuals [50]. Furthermore, metabolic changes in choline are related to a higher risk of type 2 diabetes due to impaired insulin sensitivity [51]. This may also be one of the factors related to higher mortality and aggravation due to COVID-19 in elderly, hypertensive, and diabetic patients.

When we compared the HCN and NCN groups, no significant differences were observed within their metabolomic profiles, which may be indicative that COVID-19 infection is a contributing factor to metabolic dysregulation.

## 4. Materials and Methods

### 4.1. Study Design

In view of the increased morbidity–mortality risk for hypertensive patients with COVID-19, an observational, case-control study aimed to correlate the SARS-CoV-2 infection with the response to losartan treatment in hypertensive patients has been proposed. The therapeutic response was determined through the plasmatic levels of losartan and EXP3174 and the BP control, while the clinical outcomes of COVID-19 were determined through the metabolomic profile and specific clinical analyses. Patients with a positive diagnosis presented a test report that confirmed the infection, and negative patients underwent a rapid blood antibody test (Hangzhou Biotest Biotech Co., Hangzhou, China) prior to the group distribution to confirm the result.

### 4.2. Participants and Ethical Considerations

A total of 75 patients over 18 years old were recruited between May and July 2020 and divided into four groups described below: hypertensive patients using losartan previously diagnosed with COVID-19 (HCP); normotensive patients previously diagnosed with COVID-19 (NCP), both after at least one month before participation; hypertensive patients using losartan (HCN); and normotensive patients with a negative result for COVID-19 (NCN).

Patients carrying hematological diseases, metabolic disorders, or who had donated blood in the 3 months prior to their participation were excluded. The selected participants expressed their participation by signing a consent form (ICF). The research project was submitted and approved by the Ethics Committee of the Federal University of Amapá—CEP/UNIFAP under approval number CAAE nº 37806920.2.0000.0003.

### 4.3. Determination of Plasmatic Levels of Losartan and EXP3174

Hypertensive participants received a dose of losartan of 25, 50, or 100 mg (according to the usual prescribed treatment) of Aradois^®^ from the same batch, administered with 100 mL of natural water.

Blood samples (5 mL) from hypertensive patients were collected at pre-dose (0 h) and after 1.5 and 3 h, following the expected C_max_ of losartan and its active metabolite [17, 20]. Normotensive patients in both groups (NCP and NCN) had a single collection. Samples were centrifuged at 3000 rpm for 10 min.

Analytical standards for losartan, losartan carboxylic acid (EXP3174), and irbesartan (internal standard) were obtained from Synfine Research, Inc. (Richmond Hill, Canada). Gradient and ultrapure HPLC acetonitrile were obtained from Merck (Madrid, Spain).

An Ultra Efficiency Liquid Chromatograph (Agilent, Santa Clara, CA, USA) was used, followed by a timsTOF mass spectrometer (Bruker, Billerica, MA, USA), with chemical ionization at atmospheric pressure (APCI atmospheric pressure chemical ionization), in positive mode and with a time-of-flight (TOF) analyzer. The analyses were carried out at the Scientific and Technological Support Center of the University of Santiago de Compostela, Spain.

Before the analysis, sample preparation followed protein precipitation protocols. Plasma obtained from patients was enriched with the internal standard irbesartan. Analytes were extracted from 300 μL of plasma, which was mixed with 340 μL of acetonitrile (HPLC grade) and centrifuged for 10 min at 3000 rpm at 4 °C. The supernatant layer was filtered through a syringe filter (0.22 μm) and injected into the equipment.

The analytes were extracted from 300 μL of plasma, according to the methodology previously described [52]. A 2 μL aliquot of the obtained solution was injected into the LC-MS/MS system using a C18 column at 40 °C and mobile phases: 0.2% formic acid in water and acetonitrile. A chromatographic method (LC-MS/MS), previously validated [52] in accordance with ANVISA (Brazilian Sanitary Agency) resolution 166/2017, was used for the plasmatic quantification of losartan and EXP3174, in the range of 10 to 1500 ng/mL.

### 4.4. Sample Preparation for Metabolomics and Acquisition of ^1^H NMR Spectra

Samples for metabolomic analysis were prepared according to the method used by Dona et al., 2014 [21]. An aliquot of 300 μL of pre-dose plasma and 300 μL of Na_2_HPO_4_·7H_2_O buffer were homogenized and centrifuged at 10,000 rpm for 10 min, and the supernatant was collected to measure the NMR spectra determination.

NMR experiments were conducted at 25 °C on a Bruker NEO 17.6 T spectrometer (proton resonance 750 MHz), equipped with a ^1^H-19F/13C/15N triple resonance PA-TXI probe with deuterium lock channel and shielded PFG z-gradient. The spectrometer control software was TopSpin 4.x. The chemical shifts reported are referenced to the TSP signal (δ_TSP_ = 0 ppm). Spectra were processed and analyzed with Mestrenova software v14.0 (Mestrelab Inc., Santiago de Compostela, Spain).

Proton spin-echo transversal relaxation filtered spectra (T_2_-filter) were measured with the Carr–Purcell–Meiboom–Gill (CPMG) sequence with presaturation (sequence cpmgpr1d of the Bruker library). The presaturation was applied to the water signal with a continuous wave pulse of low power and duration, 4 s. The spectrum was acquired with 128 scans, and the duration of the CPMG filter was set to 76.8 ms with an interpulse delay of 1.4 ms. The FID acquisition time (aq) was 3.14 s at spectral width of 13.9 ppm. The spectra were aligned, and the baseline was corrected. Each spectrum was bucket-integrated in segments of equal width ((Δδ) 0.04 ppm) between 0.8 and 9.23 ppm, excluding the solvent region between 4.6 to 5.1 ppm. The total integral of the buckets was normalized by the total sum.

The identification of metabolites from the NMR spectrum was performed initially with ChenoMX NMR Suite v8.5 software (Chenomx, Inc., Edmonton, AB, Canada) by comparing relevant peaks in the experimental ^1^H_T_2_ spectrum with those from the NMR database of metabolites of this software. From the set of plausible metabolites identified with ChenoMX, only those with relative abundance in plasm over 20 μM, according to the concentration reported in the Human Metabolome Database [53], detected by our NMR spectrometer were further considered.

### 4.5. Analysis of Clinical and Laboratory Parameters

In addition to blood collection for the metabolomic study of losartan, conventional analytical tests were used performed to quantify albumin, creatinine, (lactic dehydrogenase) LDH, (aspartate aminotransferase) TGO, (alanine aminotransferase) TGP, glucose, insulin, C-reactive protein (CRP), and complete blood count. Body composition analysis was also performed using bioimpedance, with the aid of a digital scale (Omron Healthcare Co., Kyoto, Japan), obtaining weight, height, body mass index (BMI), % of body and visceral fat, % of skeletal muscle, and biological age. BP was measured using an automatic arm BP meter (Omron Healthcare Co., Kyoto, Japan).

### 4.6. Metabolic Pathway

To assess the biological roles and identify overrepresented pathways in the metabolite list, the web-based Metaboanalyst 3.0 https://www.metaboanalyst.ca/ (Accessed on 1 March 2023) of and the Metabolic Biological Role (MBRole) https://csbg.cnb.csic.es/mbrole2/ (Accessed on 1 March 2023) were used. These are online platforms for metabolite annotation enrichment analysis that calculate *p*-values with the cumulative hypergeometric distribution by comparing the number of compounds in the set and in the background with a given annotation [54]. Values of *p* < 0.05 were considered significant. The Human Metabolome Database, Biological Magnetic Resonance, Bruker’s Biofluid Reference Compound Database Library, and Metaboanalyst 4.0 software were used to confirm the identity, the metabolomic pattern, and its correlation with the pharmacotoxicological parameters of losartan and its active metabolite.

### 4.7. Statistical Analysis

The data were processed using the orthogonal signal correction technique (OSC) and analyzed using Statistica 9.0 software. The O-PLS model was applied to correlate the data to the identification of biomarkers. A random permutation test with 2000 permutations was performed with the derived OPLS-DA model to confirm the robustness of the method, resulting in the reported values of R^2^ and Q^2^. Cross-validation was applied to the OPLS-DA model obtained to determine its accuracy. The bar graphs of the NMR intensities of each selected bucket were represented, and the *p*-value of the distribution was calculated with the unpaired *t*-test. The normality of the data was tested with the Kolmogorov–Smirnov normality test. If data underwent the Gaussian distribution performed with Welch’s correction, it was assumed that the standard deviation was not equal, or otherwise, the Mann–Whitney test was used.

The plasmatic levels were assessed in pairs and submitted to analysis of variance (ANOVA), followed by Tukey’s post-test. The numerical values obtained from the metabolomic analysis were processed by the orthogonal signal correction (OSC) technique [55] and also analyzed with the Metaboanalyst 3.0. The O-PLS model was applied to correlate NMR data with biomarker identification.

The Human Metabolome Database, Chenomix, Biological Magnetic Resonance and Bruker Biofluid Reference Compound Database Library, and the Metaboanalyst 4.0 software were used to confirm the metabolomic pattern and its correlation with COVID-19 and with the pharmacological parameters of losartan and its active metabolite.

Data from laboratory results, bioimpedance, BP, and questionnaires were tabulated in Excel, subjected to descriptive analysis, and correlated to the findings of the analysis of the patients’ plasma metabolomic profiles and also to the plasmatic levels of losartan and its active metabolite (EXP3174) and BP measurements.

## 5. Conclusions

NMR plasma metabolomics of hypertensive and normotensive volunteers with and without a previous diagnosis of COVID-19 identified several biomarkers capable of distinguishing these groups. Glucose was elevated in the HCP group when compared to the NCP group, which was not observed when comparing the HCN and NCN groups, indicating that hypertension together with COVID-19 contributes to this aspect and that this metabolite probably plays a major role in the pathophysiology of COVID-19 in hypertensive patients.

Furthermore, some metabolites related to inflammation, kidney damage, metabolic alterations, and persistent pulmonary dysfunction were found, indicating that the damage caused by COVID-19, especially when associated with hypertension, is difficult to recover from.

The plasmatic levels of losartan and EXP3174 were below the expected therapeutic levels, and monitoring is a key aspect, especially in patients affected by COVID-19. Moreover, COVID-19 did not affect the plasmatic levels found. From the proposed methodology, clinical protocols can be created for the therapeutic follow-up of losartan to aid in pharmacological treatments.

Part of the normotensive group (28%) presented elevated BP values, which may be an indication that COVID-19 is a presiding factor for hypertension progression.

Pharmacometabolomic has been considered a valuable resource to better understand host metabolic responses associated with COVID-19, broadening our understanding of pathogenesis in patients with different symptomatic conditions and contributing to the identification of disease biomarkers and development of tests, diagnostics, and possible strategic therapies. The results presented in this study may serve as a base for population-based studies to confirm and expand the knowledge on the relationship between COVID-19 and hypertension and anti-hypertensive treatments.

The metabolomic strategies used to combat the pandemic can form the basis for long-term planning. Further advances through these approaches will not only help in the fight against the pandemic but will also drive the wider adoption of these technologies by the scientific community and government agencies.

## Figures and Tables

**Table 1:** Hypertensive and normotensive patients’ number and subdivision.

Groups	3 Months	6 Months	1 Year	Total
HCP	*n* = 6	*n* = 4	*n* = 10	*n* = 20
HCN	*	*	*	*n* = 17
NCP	*n* = 8	*n* = 5	*n* = 5	*n* = 18
NCN	*	*	*	*n* = 20

* Does not apply.

**Table 2:** Clinical variables from recruited hypertensive and normotensive patients.

Variables	HCP (*n* = 20)	HCN (*n* = 17)	NCP (*n* = 18)	NCN (*n* = 20)
Age	52 ± 6.1	54 ± 11.1	44 ± 10.6	45 ± 9.1
Gender	M = 7 F = 13	M = 8 F = 9	M = 9 F = 9	M = 8 F = 12
Other diseases	8	7	7	4
Patients with altered blood pressure ^1^				
*Fasting*	55% ^3^	58% ^3^	28% ^3^	0% ^3^
*After 1 h 30 m*	30% ^3^	53% ^3^	*	*
*After 3 h*	20% ^3^	41% ^3^	*	*
Vaccinated with at least one dose	90%	94%	50%	90%
LDH ^2^	25%	5.8%	^a^	^a^
Glucose ^2^	75%	35%	61.1%	40%
C-Reactive protein (PCR) ^2^	55%	23.5%	5.5%	20%

¹ Reference for normal blood pressure values: systolic pressure ≥ 140 mmHg and diastolic pressure ≥ 90 mmHg. ^2^ Reference values of biochemical markers for adults: LDH—200 to 480 U/L; Glucose—70 to 99 mg/dL; PCR up to 6 mg/L. ^3^ Percentage of patients who presented high blood pressure at each measurement. * Does not apply. ^a^ Results are within the normal values.

## Data Availability

Not applicable.

## Supplementary Materials

The following supporting information can be downloaded at: https://www.mdpi.com/article/10.3390/ph16091290/s1. Table S1: Plasmatic levels of losartan and EXP3174 in COVID-19 positive and negative hypertensive patients.
