# Supplementary material for: Metabolomic Profile, Plasmatic Levels of Losartan and EXP3174, Blood Pressure Control in Hypertensive Patients and Their Correlation with COVID-19
Source: Pharmaceuticals (Basel). 2023 Sep 13;16(9):1290. doi: 10.3390/ph16091290 (PMC10535928; doi:10.3390/ph16091290)
Supplement: Supplementary file 1 [file pharmaceuticals-16-01290-s001.zip › pharmaceuticals-2488448-supplementary.pdf]

Table S1. Plasmatic levels of losartan and EXP3174 in COVID-19 positive and negative hypertensive patients.

Patient sample	Losartan(ng/mL)			EXP3174 (ng/mL)		
HCP	Fast	After 1h30m	After 3h	Fast	After 1h30m	After 3h
HCP1	62	-	20	32	14	138
HCP2	84	-	10	30	4	122
HCP3	-	30	12	-	40	144
HCP4	-	38	44	16	76	102
HCP5	-	82	46	-	20	134
HCP6	-	72	34	-	26	266[1]
HCP7	-	132	44	-	212[1]	80
HCP8	-	88	20	4	38	94
HCP9	-	42	18	10	26	160
HCP10	-	50	52	12	88	128
HCP11	-	110	44	12	60	240[1]
HCP12	6	114	16	172	244[1]	106
HCP13	-	36	36	-	46	20
HCP14	-	-	42	12	10	146
HCP15	-	36	70	-	30	208[1]
HCP16	-	-	84	-	-	348[1]
HCP17	-	106	54	26	92	138
HCP18	-	76	16	-	44	88
HCP19	-	66	34	-	54	362[1]
HCP20	2	96	20	26	318[1]	138
HCN	Fast	After 1h30m	After 3h	Fast	After 1h30m	After 3h
HCN1	-	48	-	-	44	174
HCN2	-	70	20	-	22	36
HCN3	-	40	14	-	12	102
HCN4	-	54	26	8	40	194
HCN5	-	-	20	112	22	96
HCN6	-	80	32	-	14	274[1]
HCN7	10	82	12	-	226[1]	38
HCN8	-	-	132	-	-	126
HCN9	-	-	30	26	118	138
HCN10	-	100	22	-	14	240[1]
HCN11	-	72	54	14	48	56
HCN12	-	152	-	8	72	-
HCN13	-	164	38	2	10	404[1]
HCN14	-	62	-	-	-	-
HCN15	5	-	72	210[1]	444[1]	88
HCN16	-	178	-	-	-	174
HCN17	74	-	-	78	334[1]	36

The results were calculated using the calibration curve and expressed as mean (in µg/L). Losartan EC50: 32 µg/L. Therapeutic range of losartan: 200 - 650 µg/L and EXP3174: 200 - 1200 µg/L; - Not detected; [1]Therapeutic reached the therapeutic window.
